# Evidence of Insulin Resistance and Other Metabolic Alterations in Boys with Duchenne or Becker Muscular Dystrophy

**DOI:** 10.1155/2015/867273

**Published:** 2015-05-19

**Authors:** Maricela Rodríguez-Cruz, Raúl Sanchez, Rosa E. Escobar, Oriana del Rocío Cruz-Guzmán, Mardia López-Alarcón, Mariela Bernabe García, Ramón Coral-Vázquez, Guadalupe Matute, Ana Claudia Velázquez Wong

**Affiliations:** ^1^Laboratorio de Biología Molecular, Unidad de Investigación Médica en Nutrición, Hospital de Pediatría, Centro Médico Nacional Siglo XXI, IMSS, Apartado Postal C-029 C. S.P.I. “Coahuila”, Coahuila No. 5, Colonia Roma, 06703 México, DF, Mexico; ^2^Servicio de Electrodiagnóstico y Distrofia Muscular, Instituto Nacional de la Rehabilitación, México, DF, Mexico; ^3^Sección de Estudios de Posgrado e Investigación, Escuela Superior de Medicina, Instituto Politécnico Nacional, Plan de San Luis y Díaz Mirón s/n, Col. Casco de Santo Tomas, Delegación Miguel Hidalgo, 11340 México City, Mexico; ^4^Subdirección de Enseñanza e Investigación, Centro Médico Nacional 20 de Noviembre, Instituto de Seguridad y Servicios Sociales de los Trabajadores del Estado, San Lorenzo 502 (2 Piso), Colonia Del Valle, Delegación Benito Juárez, 03100 México City, Mexico; ^5^Unidad de Investigación Médica en Genética Humana, Hospital de Pediatría, Centro Médico Nacional Siglo XXI, IMSS, México, DF, Mexico

## Abstract

*Aim*. Our aim was (1) to determine the frequency of insulin resistance (IR) in patients with Duchenne/Becker muscular dystrophy (DMD/BMD), (2) to identify deleted exons of DMD gene associated with obesity and IR, and (3) to explore some likely molecular mechanisms leading to IR. *Materials and Methods*. In 66 patients with DMD/BMD without corticosteroids treatment, IR, obesity, and body fat mass were evaluated. Molecules involved in glucose metabolism were analyzed in muscle biopsies. Results show that 18.3%, 22.7%, and 68% were underweight, overweight, or obese, and with high adiposity, respectively; 48.5% and 36.4% presented hyperinsulinemia and IR, respectively. Underweight patients (27.3%) exhibited hyperinsulinemia and IR. Carriers of deletions in exons 45 (OR = 9.32; 95% CI = 1.16–74.69) and 50 (OR = 8.73; 95% CI = 1.17–65.10) from DMD gene presented higher risk for IR than noncarriers. We observed a greater staining of cytoplasmic aggregates for GLUT4 in muscle biopsies than healthy muscle tissue. *Conclusion*. Obesity, hyperinsulinemia, and IR were observed in DMD/BMD patients and are independent of corticosteroids treatment. Carriers of deletion in exons 45 or 50 from DMD gene are at risk for developing IR. It is suggested that alteration in GLUT4 in muscle fibers from DMD patients could be involved in IR.

## 1. Introduction

Duchenne muscular dystrophy (DMD) is a recessive X-chromosome-linked disease that affects ~1/3600–6000 liveborn males. DMD usually presents in early childhood with generalized motor delays and gait difficulties. The muscular weakness is progressive, causing loss of ambulation by early adolescence (between 9 and 12 years of age) [1]. DMD is caused by mutations in the* DMD* gene that code for dystrophin, a sarcolemmal cytoskeletal protein [2–4].* DMD* gene mutations that result in complete loss of dystrophin interrupt their translation, giving rise to DMD. Mutations that conserve the open reading frame produce reduced quantities of dystrophin or a dysfunctional or truncated form of dystrophy, resulting in Becker muscular dystrophy (BMD), a less severe phenotype [5].

Dystrophin is an element of the dystrophin-glycoprotein complex (DGC) that provides a mechanical link between the extracellular matrix and cytoskeleton of muscle cells, allowing the plasma membrane of the muscle fiber to resist the mechanical process of the muscle during muscle contraction [6, 7]. It has been suggested that the DGC also participates in important cell-signalling processes, functioning as a binding platform for certain ligands [8] such as neuronal nitric acid synthase, which stimulates glucose transport [9]. Skeletal muscle is responsible for >80% of insulin-stimulated glucose uptake in the body [10]. Therefore, destabilization in DGC or their components may generate abnormal signalling of insulin in muscle fibers and lead to alterations in functionality. In fact, it was reported that the disturbance within the surface DGC may contribute to insulin resistance (IR) and abnormalities characteristic of the skeletal muscle of diabetic Goto-Kakizaki rats, because an abnormal subcellular location of glucose transporter 4 (GLUT4) vesicles in muscle fiber was observed in these rats [11]. However, information on this regard from human studies is lacking. This information led us to propose that DGC alterations may generate changes in glucose metabolism such as IR in skeletal muscle of patients with DMD or BMD.

DGC modifications provoke an imbalance in plasma membranes permeability, inducing myofibers through deterioration-regeneration cycles until exhausting their repair capacity [12]. This poses muscle fibers susceptible to necrotic development and to be replaced by both fibrous connective tissue and adipose tissue [1, 13], increasing adiposity. Previous body composition studies have showed that DMD patients present greater body fat mass than similarly aged healthy subjects [13, 14]. In addition, other investigators have observed that patients with DMD develop overweight or obesity from the age of 7, reaching a frequency of >50% at 13 years of age [15].

In addition to the alteration of DGC, overweight and obesity are also risk for IR, which increases the risk for other severe morbidities such as cardiovascular disease and type 2 diabetes in DMD patients [16]. However, the concomitant effect of alterations in DGC and obesity on the risk for insulin resistance has not been properly addressed. Thus, the purposes of this investigation were to determine the frequency of IR in patients with DMD/BMD and to evaluate the association of deletions in specific regions of the* DMD* gene with obesity and IR, as well as exploring molecular mechanisms likely leading to IR, by evaluating molecules involved in glucose metabolism such as insulin receptor, insulin receptor substrate, and GLUT4 localization in muscle biopsies of DMD/BMD patients.

## 2. Subjects and Methods

### 2.1. Patients

The Institutional (Instituto Mexicano del Seguro Social) Ethics Committee approved the study prior to the start of patient recruitment. All DMD/BMD patients seen at the outpatient Electrodiagnostic Muscular Dystrophy Service at the National Institute of Rehabilitation were recruited for the cross-sectional study between January 2011 and December 2013; 117 patients (aged 4 years to <18 years) were included. Subjects with a clinical diagnosis of DMD/BMD were candidates to participate in the study, and confirmatory molecular diagnosis of dystrophy was carried out. Patients were eligible for inclusion in this study if they had a deletion in the* DMD* gene analyzed by multiplex polymerase chain reaction (MPCR). Children were not included if they received corticosteroids. None of the patients was taking medications during the study.

Parents and patients received an explanation of study fundamentals, procedures, benefits, right to confidentiality, and the right to withdraw from the study if they wished. All parents provided written informed consent in adherence with the human subjects' guidelines of the Institutional Ethics Committee.

On the day of the study, a peripheral blood sample was collected in vacutainer with and without anticoagulant in a fasting state for genomic DNA extraction from leukocytes to determine glucose and insulin, respectively. Serum samples were kept at −70°C until analysis. A medical history was obtained, and weight and height were measured. Body composition to measure adiposity was evaluated by dual-energy X-ray absorptiometry (DEXA).

### 2.2. Anthropometric Measurements

Trained personnel carried out measurements of body weight (kg) and height (mts). For subjects who were able to stand erect, height was measured with a wall-mounted stadiometer (Model 208, Seca). For subjects unable to stand erect, length was measured on a flat table with the subject supine. Subjects' weight was measured (Model BWB-700, Tanita, for ambulatory patients and model 954 Seca for wheelchair-bound patients) wearing light clothing and without shoes.

Diagnosis of overweight and obesity was obtained using body mass index (BMI), expressed as percentiles. Children with BMI ≤ 5th percentile were classified as underweight, those with BMI > 5th but <85th percentile as normal weight, those with BMI ≥ 85th but <95th percentile as overweight, and those with BMI ≥ 95th percentile as obese, in accordance with criteria established by the Centers for Disease Control and Prevention (CDC) in 2009 about BMI for children and teens (http://www.cdc.gov/healthyweight/assessing/bmi/childrens_bmi/about_childrens_bmi.html).

### 2.3. Body Composition

Body composition measurements were carried out by DEXA (Lunar Prodigy, GE Medical Systems, Madison, WI) and enCore software, v. 2004 (Lunar Corporation), was used to analyze whole-body DEXA scans. Body fat mass was considered high according to the classification as described previously [17] that used DEXA to predict % body fat mass corresponding to the BMI cutoff in male children and adolescents (3–18 years) as overweight (range: 18%–23%) and obesity (range: 24–36%).

### 2.4. Molecular Diagnosis of DMD

DNA was extracted from peripheral blood samples according to standard procedures [18]. One hundred seventeen patients were screened for deletions and duplications by MPCR with the primer sets of Chamberlain et al. [19] and Beggs et al. [20] using the MPCR kit for Human DMD/BMD set I + II (Maxim Biotech, San Francisco, CA) according to the protocol recommended by the manufacturer.

### 2.5. Biochemical Assays

Serum insulin (*μ*U/mL) was quantified utilizing a commercial kit (Linco Research, St. Louis, MO) based on radioimmunoanalysis. *A* value >12 *μ*U/mL was considered as hyperinsulinemia as was previously reported [21]. Serum glucose (mg/dL) concentration was measured by the glucose-oxidase method (Glucose-LQ, SpinReact, S.A., Girona, Spain). IR was calculated from insulin and glucose data using the homeostasis model assessment-insulin resistance (HOMA-IR) method (HOMA-IR = [fasting insulin, *μ*U/mL] *∗* [fasting glucose, mmol/L])/22.5 [22]. Values of HOMA-IR > 3.16 [23] were considered as IR.

### 2.6. Cellular Localization of Dystrophin, Insulin Receptor, Insulin Receptor Substrate, and GLUT4 Using Immunofluorescence Analysis

#### 2.6.1. Source of Human Muscle

Muscle biopsies were obtained surgically from deltoid or biceps muscle for cytological diagnosis. A portion was used for immunofluorescence analysis. In this study, we had availability for tissues from only five patients with DMD/BMD of the entire population studied. Patient ages ranged from 8 to 11 years. We also used five biopsies of gastrocnemius muscle from five healthy individuals as controls (age 44–55 years) to compare cellular localization of dystrophin, insulin receptor, insulin receptor substrate, and GLUT4. We analyzed skeletal muscle biopsy from older controls because we have not access to biopsies of muscle from healthy subjects of the same age [13]. Nevertheless, older controls allowed the main purpose to evaluate the cellular localization of these molecules in human healthy muscle.

Specific mouse monoclonal primary antibodies used were GLUT4, insulin receptor (IRe) (Santa Cruz Biotechnology, Santa Cruz, CA), and dystrophin N-terminus (Dys-N) and C-terminus (Dys-C) (Vector Laboratories, Burlingame, CA). Polyclonal antibody against insulin receptor substrate (IRS) (Santa Cruz Biotechnology) was also used.

#### 2.6.2. Immunofluorescence Assay

Skeletal muscle biopsy was isolated and rapidly frozen in liquid nitrogen-cooled isopentane. Afterwards, 7 *μ*m cryosections were prepared and added to the coverslip covered with poly-L-lysine.

Next, nonspecific binding was blocked by incubations of the cryosections with 5% bovine serum albumin in phosphate-buffered solution (PBS) for 60 min at 25°C. Tissues were then washed with PBS. Sections were incubated overnight at 4°C with the primary antibodies. Sections were then washed with PBS. Samples were incubated with Cy3-secondary antibody (goat anti-mouse, Jackson ImmunoResearch Laboratories, West Grove, PA) for 60 min at 25°C in the dark. Subsequently, samples were washed with PBS and mounted with DAPI (labeling of nuclei) and Vectashield (Vector Laboratories). Negative controls omitting the primary antibody were included. Tissues were observed under an Olympus BX60 fluorescence microscope (Olympus, Tokyo, Japan). Five areas of 1,443,520 mm^3^ were analyzed in each section. A subjective value was assigned (normal +++, decreased ++−, considerably decreased +−−, and absent − − −) to describe staining, indicating the presence of dystrophin.

### 2.7. Statistical Analysis

Statistical analysis was performed using the Minitab statistical software (Minitab 14, State College, PA). Results are presented as median (minimum, maximum); *P* value ≤0.05 was considered significant.

Pearson correlation analyses were used to evaluate associations between the most frequently deleted exons and fasting insulin, HOMA-IR, BMI, and body composition. Comparisons among nutritional status groups were conducted with one-way ANOVA and Dunnett's method as post hoc test, considering the normal nutritional status group as control. Associations between IR and nutritional status were analyzed with *χ* ^2^ analyses. Logistic regression models were carried out to identify risk factors for IR, introducing each exon deletion as a predictor and taking into account adiposity.

## 3. Results

### 3.1. Study Population

**Figure 1:**
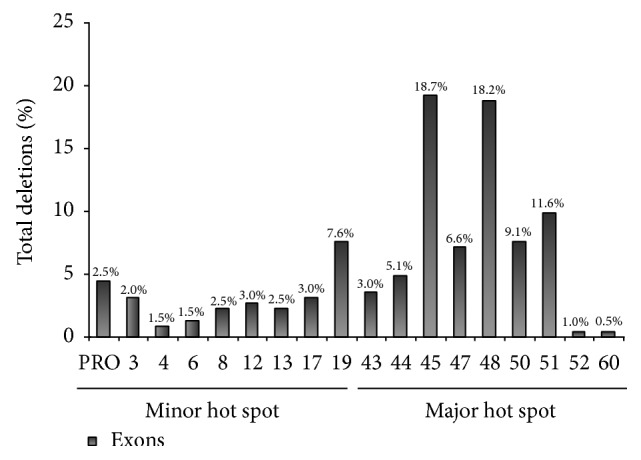
Distribution of the mutations detected in the* DMD* gene in patients with Duchenne muscular dystrophy/Becker muscular dystrophy (DMD/BMD).

We are reporting the results of 66 subjects identified as carriers in at least one exon of the two deletion-prone regions in the* DMD* gene. Most alterations (73.7%) were clustered in exons 43–60 in the major* hot spot*. Distribution of the incriminated mutations is presented in Figure 1. Deletions of exons 19, 45, 47, 48, 50, and 51 were more frequently detected.

### 3.2. Anthropometric and Metabolic Parameters

Patient ages were 8.96 (4.61, 17.75) years, median (minimum, maximum), and 53% of the population were between 7 and 10 years of age. A low percentage of patients (9.1%) were between 15 and 18 years of age. Fasting insulin concentration ranged from 5.2 to 59.9 *μ*U/mL, but ~50% present hyperinsulinemia. HOMA-IR values range from 1.04 to 12.9, and 36% of the patients had IR (Table 1). Most boys presented normal nutritional status (59%), but 22.7% were overweight/obese and 18.2% were underweight. According to the classification of Taylor et al. (see Subjects and Methods), a higher (68%) prevalence of overweight/obesity was observed in contrast to using BMI (22.7%). Thus, we show group data according to nutritional status from DMD/BMD patients as underweight, normal, and overweight/obese (Table 2).

### 3.3. Associations among IR and BMI, Body Composition, and Deletions in DMD Gene

**Figure 2:**
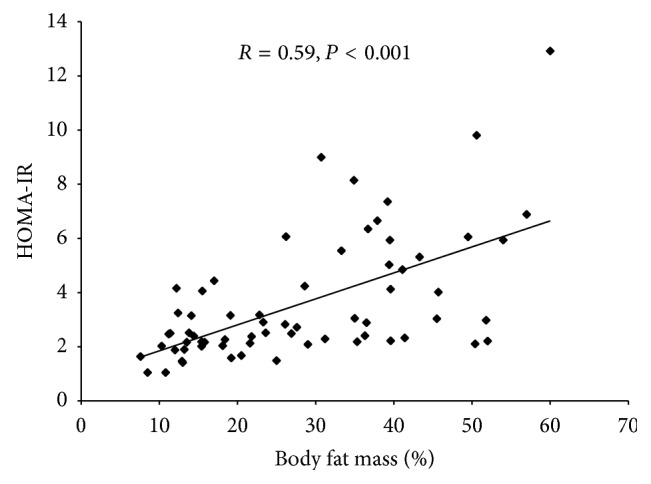
Scatter plot of body fat mass and HOMA-IR in DMD/BMD patients. HOMA-IR, homeostasis model assessment-insulin resistance.

We observed significant differences in insulin concentrations and HOMA-IR values among nutritional status groups, but no differences in age were detected. Overweight/obese boys presented higher glucose, insulin, and HOMA-IR values as compared to normal BMI group. That group exhibited also a higher proportion (80%) of subjects with IR determined by HOMA-IR > 3.16 (*χ* ^2^: 11.48, *P* = 0.004) compared to normal nutritional status. Interestingly, an important percentage of underweight patients presented hyperinsulinemia and IR (27.3%). Body fat mass was higher in overweight/obese and lower in underweight than in patients with normal nutritional status (Table 2). Both fasting insulin and HOMA-IR were positively correlated with all anthropometric and body composition parameters. However, the highest correlation was found between HOMA-IR and body fat mass (Figure 2).

**Figure 3:**
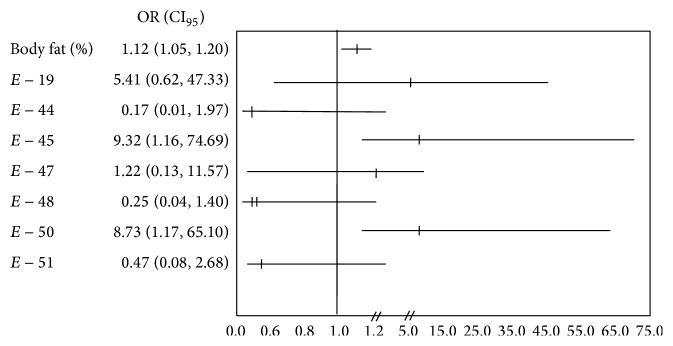
Risk of developing insulin resistance (homeostasis model assessment-insulin resistance > 3.16). *P* values and odds ratio calculated by logistic regression model. CI, confidence interval. Data adjusted by % body fat mass. *E* = Exon.

Carriers of deletions in exon 45 (OR = 9.32; 95% CI 1.16–74.69; *P* = 0.036) or exon 50 (OR = 8.73; CI_95_ = 1.17–65.10; *P* = 0.035) were associated with the risk for IR, even adjusting for adiposity (Figure 3).

### 3.4. Cellular Localization of Molecules Involved in Glucose Metabolism

**Figure 4:**
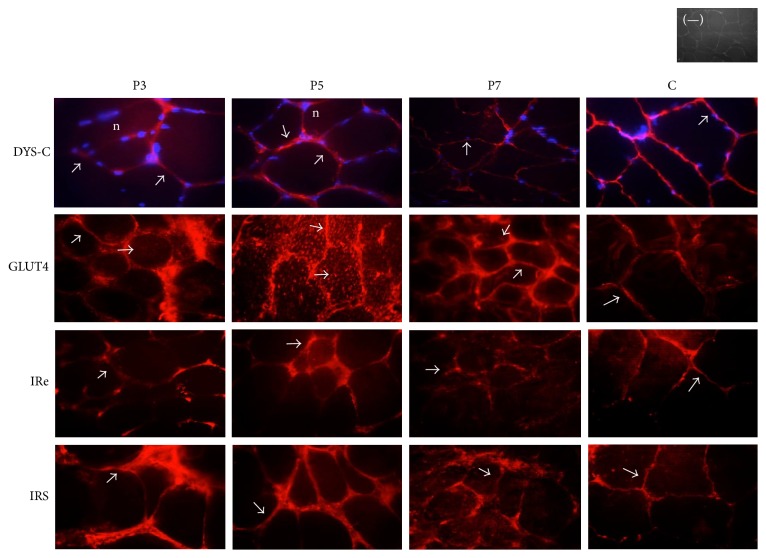
Immunofluorescence analysis of dystrophin (DYS), insulin receptor (IRe), insulin receptor substrate (IRS), and glucose transporter 4 (GLUT4) of healthy individuals and DMD/BMD patients. Immunostaining demonstrated semiabsence of dystrophin on the muscle fibers of the patients. DYS-C, GLUT4, IRe, and IRS are stained in red (arrows) and nuclei are stained in blue (n). Negative control omitted the primary antibody.

The staining of GLUT4 shows its subcellular localization in myofiber sections from muscle biopsies. Staining of GLUT4 vesicles was observed as cytoplasmic aggregates in biopsies from patients 3, 5, and 7 (Figure 4), although in patients 3 and 7 there are a smaller number of these aggregates; the three patients presented hyperinsulinemia and IR (Table 3) and patient 5 showed hyperglycemia. Aggregates were not observed in control tissue. Biopsies of remaining patients showed staining for GLUT4 similar to control tissues. Staining for IRe and IRS was similar to control tissue muscle in myofiber sections from five patients. Dystrophin staining intensities in myofibers from five patients (Figure 3) were lower than those observed in normal biopsy specimens.

## 4. Discussion

Hyperinsulinemia and IR observed in our patients are important risk factors for developing pathologies such as cardiovascular disease or type 2 diabetes. The frequency of these metabolic alterations increases (80%) importantly when DMD/BMD patients are overweight/obese, with a rate higher than in nondystrophic obese boys reported in our country (~50% in children between 3 and 18 years) [24]. The increased intramuscular body fat mass deposition [25] in DMD patients and hyperinsulinemia and IR are strong indicators of muscle metabolic defects [26]; this deposition is present in DGC-related muscular dystrophy [27]. But it is important to consider that almost a third (27.3%) of underweight patients presented hyperinsulinemia and IR which indicates that there is not an intramuscular body fat mass deposition in those patients. Therefore, IR in DMD/BMD may be an independent result of fat mass content where alterations of components of DGC such as dystrophin may be involved.

To our knowledge, there are no studies that clearly demonstrate whether patients with DMD or BMD present IR or alterations in glucose metabolism. Freidenberg and Olefsky reported that serum glucose and insulin concentrations after oral glucose administration showed an abnormal increase in the area under their respective curves, suggesting alterations in glucose metabolism from DMD patients [28].

Abnormal subcellular accumulation of GLUT4 vesicles was observed in nonobese, type 2 diabetic skeletal muscle fibers of rats [11]. Accordingly, we also observed abnormal cytoplasmic aggregates of GLUT4 in myofibers from DMD/BMD patients, suggesting a possible alteration of glucose incorporation into the muscle, leading to hyperglycemia. Interestingly, these patients also presented hyperinsulinemia and IR. It is possible that these GLUT4 abnormalities could be secondary due to IR.

Furthermore, we observed that deletions of exons 45 or 50 increase the risk (~9.0 times) for developing IR. In this sense, underweight patients who present hyperinsulinemia and IR had deletion of exons 45 or 50. These exons encode an acting-binding domain and maybe the alteration between F-actin and dystrophin link may disturb some cell-signalling process as binding platform ligands arising in metabolic alteration [8]. Anyway, our data suggest that the result of the mutation in the* DMD* gene may possibly be associated with a metabolic alteration in DMD/BMD patients. The presence of these metabolic alterations in underweight subjects suggests a possible relationship with DMD/BMD.

Because we included a low number of muscle biopsies from DMD/BMD patients with different grades of damage in the muscle fiber, we detected abnormal cytoplasmic aggregates of GLUT4 in myofibers only in three patients. At any rate, this information opens an interesting field of study to explore, in muscle biopsies from DMD/BMD patients, cellular signalling pathways involved in glucose metabolism and possibly related to muscle morphology. Another limitation of this study is the lack of distinction between DMD and BMD and the lack of appropriate control subjects of the same age. Anyway, the main strength of this study is that we demonstrated that those patients develop metabolic alterations, which is essential information for caregivers and physicians in order to prevent other pathologies.

We identified differences in nutritional status of DMD/BMD patients according to BMI (percentiles). Prevalence of overweight or obesity in these patients was lower (22.7%) than that reported by other authors where ~50% of boys with DMD/BMD were obese by 13 years of age according to BMI [13, 15]. Nevertheless, in these studies the interaction between weight and steroid treatment is noteworthy. For the first time, we present information about the prevalence of obesity in DMD without effect of steroid treatment. The higher total body fat mass observed in boys with DMD is mostly due to increased intramuscular body fat mass deposition in both the central and peripheral regions [25]. In agreement with the classification of Taylor et al., 2002, body fat mass was higher (~68%) than in healthy boys and in boys with DMD [13–15, 25].

In conclusion, to our knowledge, these novel results present evidence regarding the presence of metabolic alterations in DMD/BMD patients such as obesity, hyperinsulinemia, and IR, without the effects of steroid treatment. IR had a high frequency and increased significantly when DMD/BMD patients are overweight/obese. Deletion of exons 45 or 50 increases the risk (~9 times) for developing IR. Abnormal cytoplasmic aggregates of GLUT4 in myofibers from DMD/BMD patients suggest a possible alteration of glucose incorporation into the muscle.

## Figures and Tables

**Table 1:** Anthropometric and metabolic parameters in the study sample of DMD/BMD patients (*n* = 66).

	Median	Minimum, maximum
Age (years)	8.96	4.61, 17.75
Body weight (kg)	25.8	12.40, 79.35
Height (mts)	1.23	0.97, 1.75
BMI (kg/m^2^)	16.05	10.40, 29.50
Percentile	47.5	0.0, 99.74
Body fat (%)	26.2	7.62, 60.0
Body fat mass (kg)	6.37	0.88, 46.08
Fat-free mass (kg) Glucose (mg/dL)	16.89 91.8	10.69, 34.99 72, 135
Insulin (*μ*U/mL) >12 (*μ*U/mL)	11.75 48.5%	5.2, 59.9
HOMA-IR IR > 3.16	2.6 36.4%	1.04, 12.91
Loss of ambulation	18	

DMD/BMD: Duchene/Becker muscular dystrophy; BMI: body mass index; IR: insulin resistance; HOMA-IR: homeostasis model assessment-insulin resistance.

**Table 2:** Characteristics and metabolic variables according to nutritional status in DMD/BMD patients from 4 to 18 years of age^a^.

	Underweight	Normal	Overweight/obese
	(*n* = 12)	(*n* = 39)	(*n* = 15)
Age (years)	9.2 (4.9, 17.8)	8.9 (4.6, 17.1)	8.5 (6.2, 15.0)
Height (mts)	1.21 (0.97, 1.64)	1.26 (1.0, 1.75)	1.23 (1.15, 1.75)
Percentile of BMI	0.63 (0.0, 5.34)^*^	46.37 (6.54, 81.38)	93.2 (86.35, 99.74)^*^
Body weight (kg)	19.3 (12.4, 28.1)^*^	24.3 (14.8, 59.3)	34.7 (24.7, 79.4)^*^
BMI (kg/m^2^)	13.3 (10.4, 14.1)^*^	16.0 (13.9, 22.9)	21.3 (18.5, 29.5)^*^
Lean body mass (kg)	15.44 (10.69, 22.7)	16.97 (12.22, 34.07)	19.24 (13.76, 34.99)^*^
Body fat mass (%)	13.8 (7.6, 26.2)^*^	23.6 (10.3, 57.0)	39.6 (27.6, 60.0)^*^
Body fat mass (kg)	2.6 (0.88, 5.42)^*^	6.16 (1.58, 30.74)	13.14 (8.85, 46.08)^*^
Glucose (mg/dL)	90.08 (77.43, 104.4)	91.88 (75.67, 104.5)	97.29 (75.67, 135.12)^*^
Insulin (*μ*U/mL)	9.4 (5.2, 23.9)	11.0 (5.9, 43.5)	23.7 (9.4, 59.9)^*^
HOMA-IR	2.08 (1.04, 6.06)	2.49 (1.04, 8.99)	5.54 (2.21, 12.91)^*^
Wheelchair bound (number of boys)	2	11	5

DMD/BMD: Duchene/Becker muscular dystrophy; BMI: body mass index; HOMA-IR: homeostasis model assessment-insulin resistance.

^*∗*^ *P* < 0.01 compared to normal group (ANOVA, Dunnett's method).

^a^Values are median (minimum, maximum).

**Table 3:** Characteristics of patients and cellular localization of molecules involved in glucose metabolism.

	Patient 3	Patient 4	Patient 5	Patient 6	Patient 7
*Variable *					
Age (years)	11.2	8.07	6.2	6.48	11.2
Age of onset (years)	1.5	2	5	1.5	3
Wheelchair dependency	No	No	No	No	No
Exon deleted	48	45	45, 47, 48	44	45, 48
Dys C	+ − −	− − −	+ + +	+ − −	+ + −
Dys N	+ − −	+ − −	+ + −	+ − −	+ − −
Insulin (*μ*U/mL)	32.1	6.4	27.5	10.09	38.9
Glucose (mg/dL)	90.6	88.4	102.3	100.5	84.5
HOMA-IR	9.8	1.41	7.4	2.51	8.1

A subjective value was assigned (normal + + +, decreased + + −, considerably decreased + − −, and absent − − −) to describe staining indicating the presence of dystrophin. ND: not detected by multiplex PCR; GLUT4: glucose transporter 4; Dys-C: dystrophin C-terminus; Dys-N: dystrophin N-terminus; HOMA-IR: homeostasis model assessment-insulin resistance.
